# Protocol for a statewide randomized controlled trial to compare three training models for implementing an evidence-based treatment

**DOI:** 10.1186/s13012-015-0324-z

**Published:** 2015-09-28

**Authors:** Amy D. Herschell, David J. Kolko, Ashley T. Scudder, Sarah Taber-Thomas, Kristen F. Schaffner, Shelley A. Hiegel, Satish Iyengar, Mark Chaffin, Stanley Mrozowski

**Affiliations:** University of Pittsburgh School of Medicine, Pittsburgh, PA USA; Western Psychiatric Institute and Clinic, University of Pittsburgh School of Medicine, Pittsburgh, PA USA; University of Pittsburgh Medical Center, Pittsburgh, PA USA; University of Pittsburgh Department of Statistics, Pittsburgh, PA USA; Georgia State University School of Public Health, Atlanta, GA USA; Pennsylvania Office of Mental Health and Substance Abuse Services, Harrisburg, PA USA

**Keywords:** Implementation, Therapist training, Learning collaborative, Cascading model, Train-the-trainer, Distance education, Evidence-based treatment, Parent–Child Interaction Therapy

## Abstract

**Background:**

Evidence-based treatments (EBTs) are available for treating childhood behavioral health challenges. Despite EBTs’ potential to help children and families, they have primarily remained in university settings. Little empirical evidence exists regarding how specific, commonly used training and quality control models are effective in changing practice, achieving full implementation, and supporting positive client outcomes.

**Methods/design:**

This study (NIMH RO1 MH095750; ClinicalTrials.gov Identifier: NCT02543359), which is currently in progress, will evaluate the effectiveness of three training models (Learning Collaborative (LC), Cascading Model (CM), and Distance Education (DE)) to implement a well-established EBT , Parent-Child Interaction Therapy, in real-world, community settings. The three models differ in their costs, skill training, quality control methods, and capacity to address broader implementation challenges. The project is guided by three specific aims: (1) to build knowledge about training outcomes, (2) to build knowledge about implementation outcomes, and (3) to test the differential impact of training clinicians using LC, CM, and DE models on key client outcomes. Fifty (50) licensed psychiatric clinics across Pennsylvania were randomized to one of the three training conditions: (1) LC, (2) CM, or (3) DE. The impact of training on practice skills (*clinician level*) and implementation/sustainment outcomes (*clinic level*) are being evaluated at four timepoints coinciding with the training schedule: baseline, 6 (mid), 12 (post), and 24 months (1 year follow-up). Immediately after training begins, parent–child dyads (*client level*) are recruited from the caseloads of participating clinicians. Client outcomes are being assessed at four timepoints (pre-treatment, 1, 6, and 12 months after the pre-treatment).

**Discussion:**

This proposal builds on an ongoing initiative to implement an EBT statewide. A team of diverse stakeholders including state policy makers, payers, consumers, service providers, and academics from different, but complementary areas (e.g., public health, social work, psychiatry), has been assembled to guide the research plan by incorporating input from multidimensional perspective.

**Trial registration:**

ClinicalTrials.gov: NCT02543359

## Background

Disruptive behavior disorders (DBDs) affect a substantial number of young children, have lifelong implications if left untreated (e.g., [1–7]), and represent the most common presenting problem to community mental health centers [8, 9]. Meta-analytic reviews of treatment outcomes for DBDs (e.g., [10]) demonstrate that there are EBTs for DBDs. Parent–Child Interaction Therapy (PCIT) is a nationally recognized EBT for families who have children with DBDs [11]. The program is unique in comparison to other EBTs for DBDs in that it involves coaching parents as they interact with their young child (ages 2.5 –7 years). For each of two treatment phases, parents attend one didactic parent-only session during which the PCIT therapist teaches parents specific skills that will be “coached” in vivo in subsequent sessions. Parents attend approximately 12–20 weekly, 1-hour clinic-based sessions with their child [12].

Treatment outcome data from multiple randomized trials indicate that PCIT decreases child behavior problems, increases parent skill, and decreases parent stress [12–14]. When compared to waitlist controls, treatment effect sizes for PCIT range from 0.61 to 1.45 (absolute values) for parent report of child behavior and 0.76 to 5.67 for behavior observations of parent skill improvements [14]. Behavior observations indicate pre-post changes in parent behavior such as increased rates of praise, descriptions, reflections, and physical proximity and decreased rates of criticism and sarcasm (e.g., [15]). Parents report lower parenting stress, more internal (rather than external) locus of control, and increased confidence in parenting skills after learning PCIT. Parents report that child behavior improves from the clinical range to within normal limits on multiple, standardized parent report measures [15–17]. Studies have been conducted to understand the maintenance of treatment benefits [18–21], finding the majority of children (69 %) maintained gains on measures of child behavior and activity level, and over half (54 %) remained free of DBD diagnoses at 2 years post-treatment) [19].

The majority of PCIT treatment outcome studies have been efficacy trials. However, a number of effectiveness studies have also been conducted and demonstrated the positive impact of PCIT on parent, child, and family outcomes for families who complete treatment (e.g., [22, 23, 24 ]). These initial PCIT effectiveness studies also highlight some concerns when providing PCIT in community-based settings, such as higher rates of treatment attrition compared to efficacy trials.

Despite EBTs’ potential to help children and families, EBTs have primarily remained in university settings, with several reports (e.g., [25, 26]) highlighting a lack of access to EBTs in community settings. EBTs seem to be valued in frontline practice, and there is a strong push to implement them; however, the field seems to not know how best to do it, especially at scale. Billions of dollars have been invested in developing EBT implementation initiatives, yet EBTs have yet to reach their intended populations. Perhaps this is related to the lack of empirical attention devoted to training models for EBTs [27]. Little empirical attention has been paid to those who provide community care and how to effectively train them [28]. A comprehensive, recent review [28] found that three training models that are increasingly commonly used are Learning Collaborative (LC), Cascading Model (CM), and Distance Education (DE).

With the support of substantial federal funds (i.e., budgets of $29 (FY07) and $33 million (FY08)), The National Child Traumatic Stress Network (NCTSN) has focused on the implementation of EBTs [27] via the LC model. The LC approach was modeled after the Institute for Healthcare Improvement’s Breakthrough Series Collaborative Model [29, 30] for use in mental health [31]. LCs target and include multiple levels within an organization (clinicians, supervisors, senior leaders) by structuring information for specific roles. LCs can involve episodic meetings to share implementation tactics, plans, and evaluation results across multiple organizations involving several staff from each. The LC model has been implemented within healthcare for a variety of purposes (e.g., [32–36]). Within mental health, the NCTSN has used the LC method for a decade to implement several EBTs across the USA; however, there is only one published study on these efforts [37]. Four studies have been published on the use of a LC to improve engagement in mental health services [38–41] which provide primary support for initiating [38] and sustaining [39] gains in initial appointment show rates. Only one randomized controlled trial (RCT) has been completed with LCs [42], which did not find favorable results (the remaining used pre-post designs). When 44 primary care clinics were randomized to either a LC or control condition, there were few statistically and no clinically significant differences in preventive service delivery rates [42]. LCs likely are costly to implement because of the staff and coordination time required.

Cascading training models (also commonly called train-the-trainer models) have the potential to be time- and cost-effective and are widely used in mental health [43, 44], addictions [45, 46], medicine [47–53], and prevention [54–60]; however, this method has received little rigorous examination. The CM involves an EBT expert providing extensive clinical training to a community-based clinician who in turn replicates that clinical training with other clinicians within her organization. Within mental health, three early studies, two single subjects [61, 62], and one [63] quasi-experimental design, indicate a “watering down” effect from supervisors to staff [62]. In the only published RCT including CM (compared to expert and self-study) few differences were found between CM and expert training on fidelity or competence in rated client sessions. In role-played sessions, participants in the expert training condition evidenced greater gains initially; by 12-week follow-up, there were no differences across conditions in client or role-play sessions [46]. Train-the-trainer models may rely heavily on the individual trainer, making them vulnerable to that person’s turnover or change of role. The trainer may serve as a local champion for the model, potentially troubleshooting the implementations of organizational needs but not to the degree inherent in the LC structure.

DE, with strategies that include an individual’s attempt to acquire information or skills by independently interacting with training materials (e.g., computer, videotape review; not simply reading materials), are a common way clinicians learn new treatments [64], have been rated favorably by learners [65] and found to be a cost-effective method to increase knowledge [66, 67]. However, when stringent assessment methods are used, DE has been found to work only for some therapists (e.g., [68]) and to be only slightly more effective than reading written materials at improving knowledge [66, 69]. Of note, when more sophisticated online training methods are used, DE has shown favorable effects as compared to written materials or workshop training with regard to increased knowledge, competence, and fidelity. Sophisticated DE methods have the advantage of being cost-effective and the potential to make a broader public health impact given that more clinicians could access an online system than could attend in-person training. However, they may be less able to address implementation challenges beyond practitioner skill training, which LCs specifically and directly address and which CM models may indirectly address via a local trainer as EBT champion within an organization. In sum, each of these models has a distinct balance of potential strengths and limitations across dimensions of cost, effort, organizational impact, resilience to turnover, and sustainment.

## Methods/design

Within implementation science most conceptual frameworks acknowledge that implementation is a complex, interactive process in which clinician behavior (clinical practice) is influenced by individual and environmental characteristics as well as the quality of the intervention and the training design [70]. In this application (ClinicalTrials.gov Identifier: NCT02543359), we borrow from two frameworks: (1) the training transfer conceptual model [71] and (2) draft model of implementation research [72]. These frameworks were chosen because of their focus on training and transfer rather than broader competing frameworks.

Originally proposed by Baldwin and Ford [73], Ford and Weissbein [71] updated a conceptual model, the Training Transfer Conceptual Model, that was developed from an industrial/organizational psychology research review to articulate the conditions (training inputs) that impact training outcomes and training transfer (implementation). While the studies reviewed in these articles were not therapist/therapy studies, the conceptualization remains relevant. The review confirmed three types of training input factors that impact learning, retention, generalization, and maintenance of skills: trainee characteristics, training design, and work environment. In this study, the experimental manipulation has been at the Training Design level. The outputs of learning and retention are operationalized to include knowledge, skills, and attitude [74]. Transfer conditions include generalization and maintenance, which in this case, would include how clinicians apply the EBT in their setting and with families they treat (i.e., implementation and client outcomes).

Proctor and colleagues [72] have proposed a heuristic model that accounts for intervention strategies (EBT), implementation strategies (i.e., training models), and three distinct but interrelated set of outcomes within implementation research: implementation, service, and client outcomes. Using this taxonomy, we will use one intervention strategy (PCIT) to test three different training designs (LC, CM, DE) and will measure implementation and client outcomes to understand the conditions of transfer (generalization and maintenance). To understand the effectiveness of training methods, we will also measure training outcomes (outputs) articulated by Ford and Weissbein [71]. Measuring both implementation and client outcomes will help us to understand the success of the training condition for implementing an EBT in community settings [75]. Training outcomes (clinician level) are assessed using observational and self-report methods at four timepoints coinciding with the training schedule: baseline, 6 (mid), 12 (post), and 24 months (1 year follow-up). Immediately after clinician training began, parent–child dyads are recruited from the caseloads of participating clinicians. Client outcomes (parent–child dyad level) are assessed by parent report at four timepoints (pre-treatment, 1, 6, and 12 months after the pre-treatment). Implementation outcomes [75] (clinic level) are assessed using behavior observation, interview, and self-report measures with administrators, clinicians, and families at baseline, 6 (mid), 12 (post), and 24 months (1 year follow-up). This will include examining the overall penetration of the EBT (e.g., how extensively it is used) and whether the EBT is able to be diffused through the organization and sustained over the course of the study.

### Participants and enrollment procedures

#### Clinics

**Fig. 1:**
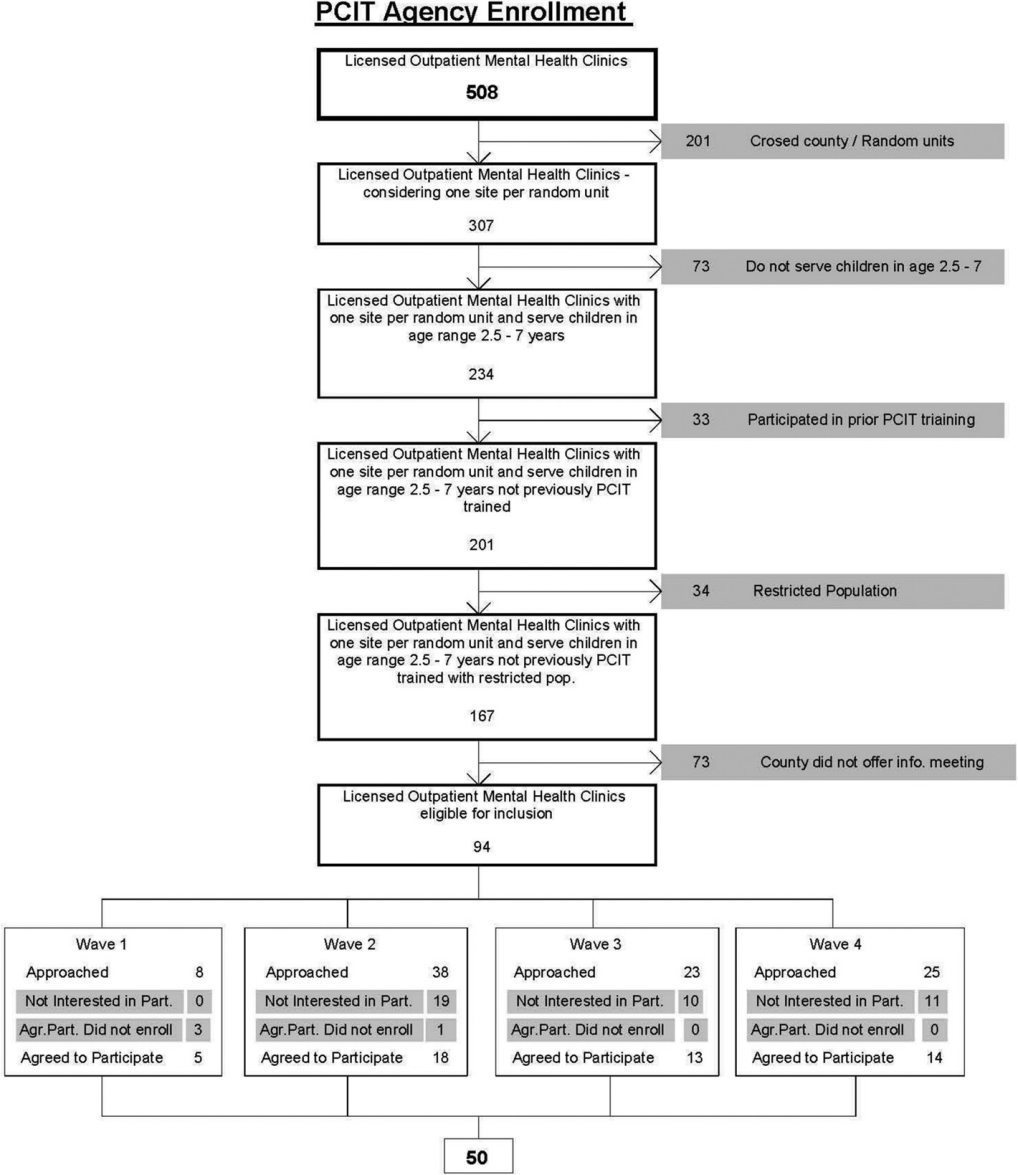
Clinic enrollment flowchart

Highlighted in Fig. 1 (clinical enrollment flowchart), there are 508 licensed, psychiatric outpatient clinics across Pennsylvania. Given concerns about contamination across study condition, if one organization operated in multiple counties (e.g., an organization had outpatient clinics in neighboring counties), the organization was only able to participate in one county. This eliminated 201 clinics from inclusion. We excluded clinics that did not treat young children (eliminating 73 clinics), had previously participated in PCIT training (eliminating 33 clinics), and had a restricted service population (e.g., specialized in treating developmental disabilities or trauma; eliminating 34 clinics).

Because we randomized at the county level, we had to approach county administrators before clinic administrators. All 67 counties were approached; 40 agreed to informational meetings. Because 27 counties did not agree to informational meetings, 73 clinics were not given the opportunity to participate. Of the remaining 94 clinics eligible for participation, 50 agreed to participate. With the support of the state children’s mental health office, we gathered data on the clinics that were not interested in participating to understand potential selection bias (e.g., clinic size, population treated, county).

To be enrolled, clinics also met the following criteria: (a) willing to participate in PCIT training, (b) able to cover site preparation costs, and (c) agreeable to research participation.

#### Administrators

To be eligible, administrators were employed at a clinic selected to participate in training as an Executive Director, Chief Financial Officer, or other person responsible for daily operations.

#### Clinicians

To be eligible, clinicians were (a) currently employed at a clinic selected to participate in training, (b) a masters or doctoral level professional in the human services field (e.g., social work, psychology, education), (c) licensed in his/her field OR receiving supervision from a licensed individual, (d) actively seeing children and families who are appropriate for PCIT (e.g., age, behavior problem severity), (e) receptive to training but not previously trained in PCIT, and (f) amenable to study tasks (e.g., video taping, completing assessments).

Based on experience with implementation studies [76, 77] and high mental health workforce turnover [78, 79], we anticipated that few clinicians will leave the training or withdraw from the study (e.g., we had a retention rate of 96 % (187/195) for clinicians in a similar trial). However, we anticipate that up to 35 % of clinicians will leave their clinics (e.g., resign) or change jobs within their clinic during the course of the study [77]. Using an intent-to-train model, clinicians who leave their clinic will remain in the study, but we may lose some clinic and client level data unless the clinician goes to another clinic that is already participating in the study or providing PCIT. All efforts have been made to get full data, but clinic level ethical, fiscal, and logistical details (e.g., FWAs, IRB protocols) may prohibit full data collection at a new site.

#### Parent–child dyads

Clinicians were asked to refer all families on their caseload with whom they are using PCIT. Inclusion criteria include any parent–child dyad who the clinician enrolled in PCIT services. A child is excluded only if he/she is a ward of the state or living in state custody. Under the study state’s law, only the birth parent or a guardian with parental rights can provide informed consent.

Clinicians ask each parent participating in PCIT to sign a “permission to contact form.” Clinicians fax the form to the research team. A research assistant contacts the parent to confirm eligibility, review informed consent including permission to video tape, and complete the first of four assessments. Subsequent assessments are completed via phone, paper, or online (depending on parent preference).

We anticipate an 88 % retention rate based on experience in previous trials; a minimum of 253 parent–child dyads likely will complete all four assessments. We estimate that the clinician to enrolled family ratio will be approximately 1:1 though we will try to improve that ratio. In previous trials, we had estimated a 1:2 ratio; however, given clinician turnover and other challenges, 1:1 has been a more accurate estimate for recent child-focused, community-based trials [80, 81].

To enhance participant recruitment and retention, incentives have been included at each level: (1) clinics receive free training for their clinicians as well as either a small stipend ($1000) to offset initial PCIT start-up costs (e.g., bug-in-the-ear) or a PCIT package including equipment (e.g., video cameras) necessary for the research and helpful for treatment; (2) administrators receive payment for assessment completion; (3) clinicians receive free training, Continuing Education Credits, and payment for time invested in assessment completion (not training); and (4) parent–child dyads receive payment for assessment completion. Top officials from several state offices and behavioral health managed care companies in Pennsylvania have endorsed and are substantively involved in this research, which also may positively impact participation.

#### Contact and monitoring procedures

Due to data collection occurring across levels of participants at multiple timepoints, we have implemented a variety of prompting and monitoring procedures to enhance participation. Specifically, participants are provided with choices related to the method of data collection (paper, online, or phone) as well as preferred contact method. The study team has utilized email, phone, postal mail, and text messaging to communicate with participants.

### Procedures

#### Randomization

Counties with at least one participating clinic located in the county were randomized to study condition [82]. Randomization occurred at the county level for two reasons. First, the state in which the study is being conducted is a commonwealth that is “state administered and county controlled” meaning that there is considerable variability in how counties implement mental health policies. Given that this project, like any EBT implementation, will require clinics to develop new programs and possibly rely on the larger system/context of which they are a part of, there could be substantial variability for implementation across counties. For example, establishing a referral base likely will impact implementation outcomes and will be different across counties. Given that this variability would be difficult to quantify, our goal has been to balance across conditions through the randomization procedure. Second, it is not logistically or financially possible to train all clinics at once. If we were to randomize clinics to a condition it is probable that within one county, we would have multiple training conditions occurring at different times. This could affect the previous training condition, particularly for smaller counties where clinics might have close communication or even share clinicians. Similar to previous trials that have randomized at the county level [82, 83], randomization of county to condition was balanced on key covariates including population size (urban/ rural) and poverty level [84].

Training occurred through four waves because it would be impossible to train all clinics at the same time. Given these constraints, we used SAS to write a routine for the randomization. Within training wave 1, counties were randomized to one of two conditions. Within waves 2, 3, and 4, counties were randomized to one of three conditions. The two conditions implemented in wave 1 were randomly chosen from the three conditions. The reason for this change was because wave 1 occurred early in the study when recruitment was beginning, and fewer counties had agreed to participate by study deadlines. Wave 1 included 5 clinics located in 2 counties. Wave 2 included 18 clinics located in 12 counties. Wave 3 included 13 clinics located in 4 counties. Wave 4 included 14 clinics located in 12 counties.

### Training conditions

The experimental manipulation in this study was within the Training Design of the Training Transfer Conceptual Model [71]. Table 1 highlights key similarities and differences across groups. Trainers were balanced across conditions. Consistent across each training condition was treatment content (i.e., treatment manual, coding manual, and workbook) and consultation with a trainer.

**Table 1:** Condition comparison on key design features

Feature	LC	CM	DE
Primary level targeted	Multiple (senior leader, supervisor, clinician)	Clinician	Clinician
Unique features	1. Addressing multiple organizational levels within the organization that might impact implementation,	1. A “top-down,” hierarchical training approach (EBT expert to a community supervisor to trainees within the community clinician’s organization),	1. Broad public health impact,
	2. Organizing “core teams” (i.e., workgroups) within each organization,	2. Strong and specific focus on two trainees who are advanced clinicians, and	2. Easy access to interactive training program online
	3. Emphasizing cross-site sharing among teams,	3. Extensive use of in vivo skill modeling, direct practice, observation and feedback within the clinic setting.	3. Revieing materials is self-paced, independent, and can be repeated
	4. Utilizing a quality improvement process, which focuses on measuring and monitoring incremental improvements		
Ultimate goal	Create a learning organization	Focus on fidelity	Make training accessible
Trainers—number	3 + admin support	2	1
Trainers—expertise	PCIT, Program Development	PCIT	PCIT
Trainees—number per site and role within organization	2–5 members comprise a “core team.” Minimally include clinicians and supervisors.	2 senior clinicians who also treat families	Unlimited
Training group size (approx)	20 participants from 6 agencies	12 participants from 6 agencies	Unlimited
Training components/ structure	Pre-work phase	5-day initial training;	Web-based training + materials
	3 2-day Learning Sessions	2-day training 6 months after the initial training	
	Action periods between learning sessions		
Time in workshop training	6 days	7 days	None
Consultation (group)	2 h per month	2 h per month	2 h per month
Time frame	12 months	12 months	12 months
Training site	Offsite	Offsite	On-site
Videotape review and feedback	Minimum four tapes per trainee by within agency supervisor	Minimum four tapes per trainee by PCIT expert	None

#### Learning collaborative

Consistent with the Institute for Healthcare Improvement [29] and NCTSN [31] protocols, the LC involved three phases: collaborative pre-work, learning sessions, and action periods. Pre-work activities were conducted prior to a face-to-face meeting to ensure that all participants come to the learning session with similar levels of PCIT knowledge. The pre-work “launch phase” (3 months) included readings, material review, and conference calls. Learning sessions included three 2-day, face-to-face meetings over a period of 9 months. Action periods occurred between learning sessions and were characterized by plan-do-study-act cycles, use of improvement data, use of technology to support learning, team meetings, and conference calls. Four people per clinic participated in the LC as part of a “core team,” including an administrator, supervisor, and two clinicians. Each LC included multiple clinic teams, was organized by a coordinator, and supported by faculty experts in PCIT and program development (e.g., financing, marketing, encouraging referrals, mobilizing clinic support). Components that make this training condition unique include the following: (1) addressing multiple organizational levels; (2) organizing “core teams”; (3) emphasizing collaborative, cross-site sharing; and (4) utilizing a quality improvement process focused on collecting implementation targets/data. After 1 year of intensive training, agencies selected one supervisor and one clinician to participate in additional training focused on training others within their organization. The inclusion of within-organization training in the LC is consistent with the intention of the training model to embed local expertise and promote sustainability. Those clinicians not involved in within-organization training continued phone consultation with the trainer at a reduced frequency (once per month) over a 6-month period (months 12 through 18 of training).

#### Cascading model

Consistent with the protocol recommended by the PCIT International Training Committee [85], this condition consisted of 40 hours of initial face-to-face contact with a PCIT trainer, an advanced live training (16 hours ) with real cases 6 months after the initial training, and bi-weekly contact with a trainer conducted over 12 months. After the end of the 12-month intensive training, trainees participated in 6 months of ongoing consultation and training, to begin training others within their organization. Each CM training group included 8 to 12 participants (two clinicians from each organization). Components that make CM unique include the following: (1) a “top-down,” hierarchical training approach; (2) strong and specific focus on two advanced clinicians; (3) extensive use of in vivo skill modeling, direct practice, observation, and feedback within the clinic setting; and (4) capacity for clinicians to return to organization as “in-house” or “within-organization” trainers to replicate clinical training with other clinicians within the organization. CM also focuses on trainers reviewing clinicians' videetaped sessions and providing them with feedback on treatment fidelity and competence. The clinicians trained by “in-house” trainers are also included in the study so that we can understand the impact of a cascading training.

#### Distance education (DE)

An online training similar to TF-CBT Web [86] was developed by the University of California, Davis Medical Center PCIT Team (SAMHSA grant; PI: Urquiza) and used in this study as part of the DE condition. The online training consisted of 11 modules/training topics, took clinicians approximately 10 hours to complete and included written materials, vignettes, videos, and quizzes for each topic. In this DE condition, the website was augmented for each participant with copies of the PCIT Treatment manual, Dyadic Parent–Child interaction Coding System (DPICS) Manual, and DPICS Workbook. Clinicians also participated in phone consultation with a trainer consistent with other training conditions.

### Outcome measures and assessment procedures

The three primary outcomes of interest are training, implementation, and client outcomes. A review of outcomes measures across participants and timepoints is provided in Table 2.

**Table 2:** Assessment measures across timepoints and participants

Construct	Measure	Brief description	Timepoints	Participant(s)	Method
Training outputs					
Knowledge					
	The PCIT Coaches Quiz [96]	22-items to assess clinicians’ knowledge of PCIT concepts and coaching scenarios; mixed question format of multiple choice and short answer	Baseline	Supervisors	Self-report
			12 months	Clinicians	
			24 months		
Skill					
	PCIT Therapist Competency Checklist	17-item checklist of PCIT competency criteria based on established PCIT Training Guidelines	Ongoing	Completed by the trainer on clinicians	Live Behavior observation
	FIRST Coach Coding System [97]	Assesses the quality and style of a therapist’s coaching	Ongoing	Completed by the trainer on clinicians	Video review behavior observation
	Supervisor Impressions of Competence in PCIT [98]	Supervision structure, practice, content, and skill	6 months	Completed by the supervisors on clinicians	Supervisor Report of clinician behavior
			12 months		
			24 months		
Attitude					
	Training Satisfaction [99]	Satisfaction with on-site training materials, content, and trainer	During each on-site training	Supervisors	Self-report
				clinicians	
	Treatment Satisfaction	Satisfaction with the treatment, concerns or barriers to using PCIT, and suggestion for improving its relevance	6 months	Supervisors	Self-report
			12 months	Clinicians	
			24 months		
	Usage Rating Profile—Intervention [100]	35-item measure of intervention usage, including acceptability, understanding, feasibility, and system support	6 months	Supervisors	Self-report
			12 months	Clinicians	
			24 months		
	PCIT Learning Experience Feedback Form	8-item survey to assess satisfaction with PCIT training	6 months	Administrators	Self-report
			12 months		
			24 months		
Training inputs					
Trainee characteristics					
	Demographic Information Form	Basic demographic and contact information	Baseline	Administrators	Self-report
				Supervisors Clinicians	
	Treatment Provider and Practices Survey [101]	Demographic information and variables hypothesized to affect adoption of EBTs	Baseline	Supervisors Clinicians	Self-report
Training design					
	Training Fidelity Checklist	Checklist to ensure that trainers implement the LC and CM conditions in the manner intended (accuracy, consistency)	During each on-site training	Research staff member	Behavior observation/ video review
Training dose					
	Consultation Records	Standardized form completed by the trainer for contact with trainees to document key topics discussed	During each consult call	Completed by the trainer on clinicians	Trainer report
	PCIT Feedback Form	Clinical open-ended feedback provided to clinicians on clinical competency	Ongoing	Completed by the trainer on clinicians	Behavior observation/ video review
Organizational/work environment					
	Infrastructure Survey of Children’s Mental Health [102]	Structured, 1-hour, interview survey to obtain information on the governance, financing, staffing, services, and implementation practices of organizations as well as their perspectives on factors important to the implementation of new treatment and services	Baseline	Administrators	Interview (qualitative and quantitative)
			12 months		
			24 months		
	Dimensions of Organizational Readiness—Revised [103]	21-item scale that requires respondents to rate the importance of specific intra- and extra- organizational variables (e.g., leadership support, fiscal benefits, match, admin burden) likely to affect implementation	Baseline	Administrators	Self-report
			12 months		
			24 months		
	TCU Organizational Readiness for Change Survey [104]	Assessment of organizational functioning including program needs, training needs, and pressures for change, program resources, and organizational dynamics	Baseline	Administrators	Self-report
			12 months	Supervisors	
			24 months		
	TCU Survey of Organizational Functioning [104]	Includes the TCU Organizational Readiness for Change plus nine additional scales assessing job attitudes and workplace practices	Baseline	Administrators	Self-report
			12 months	Supervisors	
			24 months		
Implementation outcomes					
Treatment process and acceptability					
	Treatment Implementation Feedback Form [105]	13-item questionnaire to understand clinicians’ experiences using an EBT including the extent of use, relevance, and helpfulness of the treatment	6 months	Supervisors	Self-report
			12 months	Clinicians	
			24 months		
	Treatment Summary Report [106]	15-item questionnaire to assess nature and outcome of treatment services for a particular family (e.g., service length, barriers, disposition) to provide acceptability data for a specific family	Ongoing as families discharge from treatment	Supervisors	Clinician report and chart review
				Clinicians report on child and family behavior	
Fidelity					
	PCIT Session Fidelity Checklist [107]	Detailed checklists of components of each PCIT session to measure fidelity	Ongoing through video review	Completed by the trainer on clinicians	Behavior observation/ video review
	Use of Treatment Practices [108]	15-item clinician self-report of completion of specific treatment tasks with an individual family	Ongoing as families discharge from treatment	Supervisors	Self-report
				Clinicians	
Cost					
	Chief Financial Officer Survey (updated version of a survey created by Olmstead et al. [109])	Provides information necessary to complete the cost analysis (e.g., program overhead and fringe rates, annual expenditures	Baseline	Administrators	Self-report
Adoption	Time-tracking phone app	Provides time estimates of trainer activity by condition	Ongoing	Trainers	Self-report
	Evidence-Based Practice Survey [110]	List of evidence-based practices respondent has implemented in the last year	Baseline	Administrators	Self-report
			12 months	Supervisors	
			24 months	Clinicians	
Penetration					
	PCIT Learning Experience Feedback Form	Report of consumer levels (e.g., the number of consumers who receive PCIT divided by the number of consumers who were eligible to receive PCIT and did not receive it	6 months	Administrators	Self-report
			12 months		
			24 months		
Sustainability					
	Evidence-based Practice Sustainability Telephone Interview	Survey of current PCIT delivery, penetration, practice adaption, and barriers to sustaining PCIT practice within the organization	24 months	Administrators	Self-report
Family and child outcomes					
Family characteristics					
	Demographic Information	Measure to gather information including age, race, gender, family constellation, living arrangement, school placement, and socio-economic status.	Baseline	Families	Self-report
Child focused			s		
	Eyberg Child Behavior Inventory (ECBI) [111]	36-item parent report of child conduct problems	Baseline	Families	Self-report
			3 months		
			6 months		
			12 months		
	Vanderbilt Assessment Scale Parent Version [112]	47-item assessment of core symptoms of DSM-IV diagnostic criteria (related to inattention, hyperactivity, oppositional behaviors, conduct problems, and anxiety depression) and impairment in performance	Baseline	Families	Self-report
			3 months		
			6 months		
			12 months		
	Dadds CU Scale [113]	20-item rating scale to assess psychopathic traits (narcissism, impulsivity, callousness-unemotionality) in children ages 6-13	Baseline	Families	Self-report
			3 months		
			6 months		
			12 months		
Parent focused					
	Public Health Questionnaire—9 [114]	10-item self-report to assess and monitor symptoms consistent with Major Depressive Disorder	Baseline	Families	Self-report
			3 months		
			6 months		
			12 months		
	Generalized Anxiety Disorder Scale—7 [115]	7-item self-report scale to screen and monitor symptoms consistent with Generalized Anxiety Disorder	Baseline	Families	Self-report
			3 months		
			6 months		
			12 months		
	Alabama Parenting Questionnaire [116]	Assessment of parenting practices yielding three subscales (Positive Parenting, Inconsistent Discipline, and Poor Supervision)	Baseline	Families	Self-report
			3 months		
			6 months		
			12 months		
Treatment participation					
	Barriers to Treatment Participation Scale [117]	44-item parent report of potential barriers to treatment participation	3 months	Families	Self-report
			6 months		
			12 months		
Treatment use and satisfaction	Therapy Attitude Inventory [118]	10-item, 5-point Likert-type parent report of satisfaction with process and outcome of therapy	3 months	Families	Self-report
			6 months		
			12 months		
	Multi-Sector Service Contacts—Revised Caregiver Form [119]	Report of additional services received by family/child in past 3 months and satisfaction with service(s)	Baseline	Families	Self-report
			3 months		
			6 months		
			12 months		

### Aim 1: determine the effects of training condition (CM, LC, DE) on training outputs (i.e., clinician knowledge, skill, and attitude)

#### Analyses

We hypothesize that the greatest improvements will be found for clinicians in the CM group followed by LC, clinic staff trained by clinicians in CM and LC groups, and DE (in that order). We will use the change scores on the following Ford-Weissbein [71] training outcomes: Coaches Quiz, Competence Check, and Coach Coding. Because training is at the clinician level, we will compute the change scores for each clinician and then compare the treatments using those changes. We recognize that the clinicians are nested within organization and will include that in our regression model, along with any other covariates that are associated with outcomes. We will use the one-way analysis of variance (ANOVA) to compare the change scores between the four groups. And because we have stated an ordered alternative (e.g., CM better than LC), we will use Bartholomew’s test which is powered for ordered alternatives unlike the usual F test, which is an omnibus test. Later, we will test the cascading effect of the CM model in comparing the performance of "second generation" CM clinicians to other clinicians in other conditions (CM-generation one, DE, LC). 

### Aim 2: explore implementation outcomes, including acceptability, adoption, appropriateness, feasibility, fidelity, penetration, sustainability, and cost across training conditions

#### Analyses

Using the taxonomy of implementation outcomes defined by Proctor et al. [87], data will be collected on acceptability, adoption, appropriateness, feasibility, fidelity, penetration, and sustainability. We have four main hypotheses related to implementation outcomes. First, we hypothesize that rates of acceptability and appropriateness of PCIT will be high and equal across groups. Without doing a formal hypothesis test, we will examine the rates of acceptability and appropriateness. Second, we hypothesize that rates of adoption, fidelity, and feasibility will be the highest among the more active training conditions (LC, CM). As in Aim 1, we will compare these measures using a one-way ANOVA, with Bartholomew’s test for the ordered alternative. Third, we hypothesize that implementation costs will be lowest for the DE and highest for LC. We will compare the costs per clinician for the various treatments using ANOVA comparing the three once again. Measurement and analysis of cost will be supported by a consulting health economist. Finally, we hypothesize that participants in the LC will evidence greater penetration and sustainability of PCIT within the service settings. Because penetration is a proportion, we will first stabilize variances using the arcsin transform; we will then use one-way ANOVA as in Aim 1.

### Aim 3: evaluate improvement for parent–child dyads treated by clinicians across training conditions, and explore the influence of multi-level moderators (clinician characteristics, work environment) and mediators (fidelity) of treatment gains

#### Analyses

We hypothesize that improvements will be greater for parent–child dyads treated by clinicians in the CM condition followed by LC, clinic staff trained by clinicians in the CM group, and DE (in that order). Our basic approach will be to use ANOVA methods as in Aim 1 but with proper accounting of the nesting of dyads. We expect, on average, approximately one dyad per clinician; however, whenever we have more than one dyad for a clinician we will use the data from those dyads to estimate the associated variance component, which in turn we can use to test the fit of our model. In order to examine multi-level moderators and mediators of treatment, we will use the standard framework of Baron and Kenney [88] and as elaborated by Kraemer et al. [89] to test for moderators and mediators. We regard these analyses as exploratory because tests of interactions are often not as powerful as those for main effects.

### Proposed statistical analyses

After collecting and organizing the data, we will begin with simple graphical and numerical summaries to (for example) check for unusual observations and to assess patterns of missing data. We will then address the main aims beginning with the simplest version of the hypotheses, which in this case, will typically require an ANOVA (paying attention to the nested design) to compare training conditions. We will then do a more nuanced study of the data using appropriate (e.g., linear, logistic) regression models to relate the outcomes to both features that we used to balance the randomization, covariates such as those we balance on (population size, poverty level) and other covariates that appear to be related to differences between training conditions.

#### Nesting

An important feature of this study is that it is a nested design: the clinicians are nested within clinic, and the parent–child dyad is nested within a clinician. Thus, the models that we will use to compare outcomes across treatment groups will incorporate that feature, and tests of hypotheses will involve a careful study of the resulting variance components. As a simple example at the dyad level, consider the CBCL total outcome: the model that we will consider models is *Y*(*ijk*) = *m* + *t*(*i*) + *s*(*j*) + *c*(*k*(*j*)) + error(*ijk*), where m is the grand mean, t(i) is the effect of training condition *i*, s(j) is the effect of clinic (*k*), *c*(*k*(*j*)) is the effect of clinician *j*, who is nested in site k, and error (*ijk*) is the variation unaccounted for by the covariates in the model. We will consider the use of random effects to model the (likely presence of) correlation between measures within a clinic. To fit these models and to test our hypotheses, we will use PROC MIXED in SAS.

#### Missing data

We recognize that missing data will occur because of attrition and other reasons. We will use an intent-to-treat approach: that is, a participant (e.g., clinician or dyad) who is randomized to a particular training condition will be included in the analysis whether they drop out or not. Our general approach to missing data will follow that of Little and Rubin [90]. Because the reasons for missingness may be difficult to ascertain, we will use sensitivity analysis.

### Trial status

To date, all four waves of training have been initiated. Training efforts and clinical consultation will continue through 2016. County and clinic recruitment is now complete, with 50 clinics, representing 37 randomization units enrolled in the study, which is lower than the originally predicted enrollment of 72 clinics [91]. From participating agencies, 100 clinicians, 50 supervisors, and 50 administrators have been enrolled. Additionally, 26 “second generation” clinicians have been enrolled and will continue to be enrolled as the study progresses. At the time of the manuscript acceptance (9/16/15), 203 families had consented to participate in the study; 198 families had completed the baseline assessment. Family enrollment is expected to continue through December 31, 2016, and it is anticipated that family enrollment will surpass the target of 288. Thus far, retention rates have varied across participant types but have remained high. As indicated above, we anticipate a 96 % retention rate for professional participants; to date, 94 % of professionals have been retained. We anticipate an 88 % retention rate for families; to date, 95 % of families have been retained. Likewise, data collection thus far has yielded assessment completion rates of 93 % for professional participants and 86 % for families. A few study team decisions have influenced these rates: (1) we kept in the study all clinicians who left their original agencies and (2) (if possible) we included families from these clinicians who were seen in new agencies. We recognize that these rates currently are within or surpass the expected range; however, it is early in the study timeline, and many things likely will affect final rates.

## Discussion

### Innovation and anticipated contribution

This study offers a direct comparison of the effectiveness of three training models to implement PCIT, a well-establish EBT, within community settings. As indicated above, several reports (e.g., [25, 26]) note a lack of access to EBTs within community settings. The field’s lack of successful implementation may be related to a lack of empirical attention devoted specifically to training models. To date, the most common way to train community therapists has been to ask them to read written materials or attend workshops. There is little to no evidence that these “train and hope” approaches [92] will result in increases in skill and competence [28, 93]. Although the examined training methods are becoming more commonly used to implement EBTs in community settings, limited data exist for each training method. No trials were found that included a comparison of these training conditions, and few studies [94] have directly compared active training conditions. In order to implement each training condition, great strides were taken to study and operationalize each training protocol (e.g., [95]. This study will explore outcomes at several levels (i.e., administrators, supervisors, clinicians, and families) and across training conditions (i.e., Learning Collaborative (LC), Cascading Model (CM), and Distance Education (DE)). In turn, the study protocol and findings are anticipated to contribute substantially to the literature and field by examining the effectiveness of training practices of EBTs for professionals working in real-world, community settings, connecting training models to client outcomes, and examining broader public health implications by exploring the cost, feasibility, and far-reaching impact of each training model on community systems.

### Practical and operational issues

Although this trial offers an innovative exploration of the implementation of an EBT within community settings, this strength also brings unique challenges. The context of the current study required frequent and thoughtful consideration of the methodological challenges that real-world circumstances presented over the course of the study.

As anticipated, movement and turnover of professional participants (i.e., administrators, supervisors, and clinicians) occurred throughout the course of the study, although this movement may impact the collection of client level data; to date, the rate of staff turnover has been lower (19 % within a one year time frame) than anticipated (35 % or higher) [120, 121]. By using an intent-to-train model, the study design allows for the continued tracking of professionals, as they change employment location or status. Through this design, we are able to continually collect data on clinician use of PCIT, as well as family outcomes, regardless of staff movement. Participating professionals were not replaced at the clinic level. This allowed for data collection to remain consistent over time while providing information about the frequency and nature of staff movement occurring in community mental health settings.

Demands unrelated to the study were placed on agencies during the study timeframe. For example, agencies reported experiencing an increasing number of audits, monitoring, and site visits from payer organizations and regulating bodies. In addition, several agencies reported administrative restructuring or clinic reorganization, which often resulted in staff turnover. One clinic reported that reorganizing resulted in about 30 % of staff leaving the organization within a few weeks, which impacted the roles and demands of participating team members.

The scale of this community-based study spanning across 50 agencies in 37 mental health systems led to additional considerations specific to project implementation. For example, study commitment was variable across participating professionals, organizations, and counties and has changed over time. The nature of receiving free training and support may have impacted the engagement of professionals over time. Other organization initiatives (e.g., implementing other EBTs, developing a new program) over the course of the study may have also contributed. Several components of data collection (e.g., fidelity monitoring, evaluation of clinician, and parent skill) relied on clinicians submitting video recordings of treatment sessions, and clinicians expressed a variety of barriers to successful submission of session recordings, including difficulty with technology, lack of access to computers, limited time, and reluctance to be recorded.

Challenges also exited for our research team such as the need for experienced personnel (e.g., trainers), extensive travel, and broad scale recruitment and retention efforts made this an expensive trial. Also, an online data collection system was developed because participants were located across a large geographic area, which changed many of our team’s established recruitment and retention strategies.

## Conclusions

This trial is a novel exploration of the effectiveness of three training models (Learning Collaborative (LC), Cascading Model (CM), and Distance Education (DE)) to implement a well-established EBT in real-world, community settings. The project is guided by three specific aims: (1) to build knowledge about training outcomes, (2) to build knowledge about implementation outcomes, and (3) to understand the impact of training clinicians using LC, CM, and DE models on client outcomes. This study will provide timely and relevant information to the field of implementation science, while also contributing to the broader public health impact by providing training, support, and resources to an existing workforce of service providers as well as increasing the availability of an EBT for families served in existing communities.

## Abbreviations

CM: Cascading Model training
DBDs: disruptive behavior disorders
DE: Distance Education training
EBTs: evidence-based treatments
LC: Learning Collaborative
PCIT: Parent–Child Interaction Therapy
